# Wanted: Class VI Antiarrhythmic Drug Action; New Start for a Rational Drug Therapy

**DOI:** 10.16966/2379-769X.148

**Published:** 2018-10-04

**Authors:** Hrayr S Karagueuzian, Uwe Klein

**Affiliations:** 1 Department of Medicine, David Geffen School of Medicine, Los Angeles, California, USA; 2 Cardiovascular Research Laboratories UCLA, Los Angeles, California, USA; 3 Numerate Inc., San Francisco, California, USA

Since the discovery by a Dutch merchant in the early 20^th^ Century that the drug quinine he was taking to prevent malaria during his frequent trips to the West Indies also prevented his Atrial Fibrillation (AF) [1], drug therapy of cardiac arrhythmias remained largely empirical and at best suboptimal [2]. Slowing of impulse conduction (akin to local anesthetic effects) and increased refractoriness of cardiac tissue as observed with quinidine (quinine’s isomer) still remain the two key parameters, among other mechanisms, that are targeted to control cardiac arrhythmias.

Indeed, current Antiarrhythmic Drugs (AADs) belonging to Classes I, III and IV, according to Vaughan-Williams classification alter in one way or another, these two parameters to effectively suppress the arrhythmia. Re-entry, i.e., the re-excitation of the heart by a persisting propagating impulse following the refractory period is considered as the primary electrophysiologic mechanism for clinically important arrhythmias. While re-entry is an important mechanism of clinical arrhythmias, it is not the only mechanism that is operative for both the initiation and the maintenance of the arrhythmia, as is discussed later in this mini review. Accordingly, block of the peak Na^+^ current without changing the resting membrane potential of cardiac action potential (Class I AADs) slows conduction; block of the K^+^ ion channels (Class III AADs) results in the lengthening of the cardiac refractory period, and Class IV AADs block peak L-type Ca^2+^ channels suppressing slowly conducting fronts and reentrant pathways supported by the slow inward Ca^2+^ current [3]. Alterations of either one of these two variables provided a logical explanation for the prevention or facilitation of re-entry observed in experimental models *in vitro* [4]. Almost all the drugs in Classes I, III and IV manifest multiple ion channel blocking effects, with diverse degrees of relative potencies for block of the Na^+^, Ca^2+^ and K^+^ currents. It is thought that these multiple effects of drugs conspire to alter in one way or another the finely tuned balance between conduction velocity and refractory period that is necessary for re-entry formation, as discussed by Rosen MR, et al. [5] among the authors of the Sicilian gambit. These authors recognized that the suppression of arrhythmia mechanisms is much more complex than anticipated. In their own words: *And so, we continue to operate in a gray zone, albeit one in which there is more recognition of the complexities that face us than previously was the case*.

It must however be emphasized that to derive antiarrhythmic benefit from drug-induced changes in conduction velocity, including conduction block, it is necessary that these effects occur regionally and only during the tachycardia. These important considerations further add to the complexity and difficulty of targeting a specific arrhythmia mechanism with a specific drug action.

Regarding the other classes of AADs, Class II is for drugs with predominantly beta adrenergic receptor blocking effect and Class V contains agents with diverse actions including adenosine, ivabradine and digitalis [2]. While the reduction of conduction velocity and prolongation of the refractory period by the currently used AADs may successfully terminate re-entry, it needs to be noted, that these same effects have been shown in multiple studies to also promote pro-arrhythmic events through mechanisms that remain poorly explored and understood. The unexpected pro-arrhythmic effects of current AADs therefore failed to establish drug-induced alterations of conduction and refractoriness as reliable therapeutic end-points to control cardiac arrhythmias [6,7]. Furthermore, the Vaughan-Williams classification does not take into account abnormal ionic current mechanisms in diseased cardiac conditions, the main focus of this mini review. The lack of specific drug target(s) for the effective and reliable control of arrhythmias has greatly diminished the enthusiasm in innovative AAD research, which has remained dormant for over two decades [2,8]. As lamented by van Hamel [9]: *Manifestations of an arrhythmogenic substrate and its triggers can be almost completely suppressed for a while in clinical terms defined as therapeutic success and therefore satisfying without understanding in detail its mechanisms, anatomical extent, natural course and potential complications. This attitude towards arrhythmia management offers only short-term solutions and still worse, suppresses our intellectual curiosity*.

Indeed, one such example is the Angiotensin Receptor Blockers (ARBs) which reduce the incidence of sudden cardiac death presumably caused by ventricular fibrillation by mechanism(s) that remain ill-defined [10].

It is clear that a rational and effective AAD therapy requires both the knowledge of the specific ionic mechanisms that initiate the arrhythmia and the availability of a drug to specifically and potently target this arrhythmia-causing ionic mechanism without affecting other ionic currents. At the present however, there is no such drug available to the treating physician. The lack of a rational and specific approach to AAD therapy has perpetuated empiricism and has led to suboptimal treatment outcomes [2,5].

Recent discoveries however, made on animal models and scaling from animal and human primary or induced pluripotent stem cell (iPSC)-derived cardiomyocytes to intact heart preparations, provide novel and guiding insights that pave the way for the development of more rational and effective therapies for certain types of arrhythmias. These seminal discoveries identified a rise in the late inward Na^+^ current (I_Na-L_) and the late L-type Ca^2+^ (I_Ca-L_) currents to play a key role in promoting Ventricular Tachyarrhythmias (VT/VF) with potential relevance to human hearts with diverse pathological conditions. These two late inward currents are enhanced during pathological activation of Ca^2+^/calmodulin-dependent protein kinase II (CaMKII) signaling [11], and hypokalemia [12,13], in patients at risk of VT/VF [14], by promoting Early and Late Afterdepolarizations (EADs and DADs respectively) and triggered activity. Intact isolated-perfused heart studies in rats and rabbits demonstrated that activation of CaMKII signaling, either by oxidative stress or by hypokalemia, promotes EADs and EAD-mediated triggered activity initiating VT/VF [12,13]. Traditional AADs primarily targeting peak Na^+^ and Ca^2+^ currents (Classes I and IV) lack specificity against the I_Na-L_ and I_Ca-L_. These drugs, while minimally decreasing the late components of the inward currents potently suppress the peak Na^+^ (Class I) [15] and Ca^2+^ (Class IV) transient amplitudes [16]. Unfortunately the potent suppression of these peak currents, which are necessary for proper cardiac functioning, alter normal cardiac physiology, reduce contractility and can result in proarrhythmic events [6,7]. Clearly, the development of drugs that specifically modulate the late Na^+^ and/or the late Ca^2+^ currents, without affecting the amplitude of their peak transients, will be of immense therapeutic value for the prevention of VT/VF in hearts triggered by elevated I_Na-L_ and I_Ca-L_. We have proposed that drugs that specifically and exclusively inhibit the I_Na-L_ and I_Ca-L_ without affecting the respective peak currents, i.e., gating modifier drugs, to be termed as having “Class VI” AAD action [17] (Figure 1).

In this Mini review we argue that growing evidence justifies the addition of drugs with Class VI action to the traditional Classes I to V of AADs to improve the efficacy and specificity of the currently available treatment options that clearly remain suboptimal [17,18].

Diverse acquired cardiac pathologies at risk of developing VT/VF manifest sustained hyperactivity of cardiac CaMKII, which acts as sensor of calcium and redox signaling and enhances both the I_Na-L_ and late I_Ca-L_ [13,19–24]. Similarly, cardiomyocytes isolated from patients with the congenital long QT syndrome-3(LQT3) [25,26], and the LQT8 (Timothy Syndrome) [27–30], also manifest abnormal elevation of the I_Na-L_ and late I_Ca-L_, respectively (Figure 2). Interestingly, these studies have also shown that increases in I_Na-L_ and late ICa-L in the cardiomyocytes are associated with cellular EADs and triggered activity [18,20,31–34]. Isolated intact heart [13,20] and simulation [35] studies have shown that cellular EAD-mediated triggered activity promotes VT/VF that becomes sustained through mixed focal and reentrant activity [12,35].

Electrophysiological experiments using the dynamic-clamp technique have provided compelling evidence that the observed associations between the enhanced I_Na-L_ and late I_Ca-L_ on one hand and EADs on the other are causally related. The dynamic-clamp method allows in real time, the computation and introduction of specific and programmable ‘virtual’ currents to a cell, to alter the kinetics of I_Na_ and I_Ca-L_ activation and inactivation (gating modification), followed by determination of the effects of the introduced changes on the cell’s action potential characteristics. Using this technique of electronic pharmacology, Madhvani RV, et al. [36,37] systematically analyzed the effects of selective modification of the steady state properties of I_Ca-L_ in rabbit ventricular myocytes. They found that either a mere 5 mV depolarizing shift in the voltage dependence of activation, or a hyperpolarizing shift in the steady-state inactivation curve, or a reduction of the late non-inactivating pedestal current all result in effective suppression of EADs with a normally maintained Ca^2+^(i) transient (Figure 1). Similarly, in 2D simulated cardiac tissue we found that a mere rise of the I_Na-L_ equivalent to less than 1% of the peak I_Na_ promoted EADs and resulted in triggered activity [35].

An important step towards proof of the concept of Class VI AAD action was the discovery that the tri-substituted purine derivative roscovitine, a compound that is clinically studied as an anticancer agent, potently reduces the late I_Ca-L_ without any effect on the peak I_Ca-L_. This finding was the first experimental demonstration of an archetypical small-molecule compound with Class VI action [30]. Most interestingly, roscovitine reduced the late I_Ca-L_ in human iPSC-derived cardiomyocytes from patients with LQT8 [29,38] (Figure 2). Experimental studies in isolated myocytes and intact hearts under oxidative stress confirmed that the elevation of the late I_Ca-L_ and resulting EADs were potently suppressed by roscovitine, which restored normal action potential [39]. Most interestingly, VT/VF induced by the same stressors (oxidative stress and hypokalemia) in the isolated intact heart preparations was also powerfully suppressed by roscovitine [40]. It does need to be mentioned that Roscovitine, which is a potent inhibitor of cyclin-dependent kinases will remain a prototypical example, and will not be clinically useful as an AAD therapy due to its inherent mechanism-based safety and tolerability liabilities stemming from kinase inhibition [41].

In addition to the discovery of roscovitine as a first archetypical member of Class VI AADs, dynamic-clamp experiments have led to the discovery of a second approach to novel gating modifiers that suppress EADs by narrowing the Ca^2+^ window current. An increase in the Ca^2+^ window current, i.e., the intersection of steady-state activation and steady-state inactivation curves (Figure 3) facilitates the formation of EADs [36]. Consequently, drugs that narrow the voltage range of the Ca^2+^ window current by either shifting the steady-state activation curve towards the depolarizing direction or by shifting the steady-state inactivation curve towards the hyperpolarizing direction should prevent the emergence of EADs (Figure 1). Indeed, Savalli, et al. [42] in preliminary studies showed that the gabapentinoid class of compounds exerts a shift of the steady-state activation curve towards the depolarizing direction. The two gabapentinoids gabapentin and pregabalin, drugs that are clinically used for the treatment of neuropathic pain and epilepsy, selectively bind to the α_2_δ−1 CaV auxiliary subunit, resulting in modulation of gating by shifting the voltage-dependence of steady-state activation. This slight voltage shift in steady state activation reduced the window current and prevented EADs in isolated cardiomyocytes and EAD-mediated VT/VF in intact hearts. Existing Ca^2+^ channel blockers (Class IV antiarrhythmics, like Verapamil) in addition of blocking the late I_Ca-L_ potently block the peak I_Ca-L_, thereby reducing cardiac contractility, which is contraindicated in many patients at risk for VT/VF, limiting their widespread clinical use. The gabapentinoids as gating modulators therefore represent the second prototypical entry to the Class VI AAD category, with clearly differentiated pharmacological action and effects on cardiac function relative to the traditionally used Ca^2+^ channel blockers. The centrally mediated side effects (dizziness, drowsiness, loss of balance or coordination) of the gabapentinoids however hamper their possible clinical use as ADDs, and different compounds devoid of central action need to be discovered and developed, to realize the potential therapeutic benefits of this new class.

As a third entry to the Class VI AAD category, recent investigations succeeded in the discovery of novel small molecule gating modifiers that selectively and exclusively block the I_Na-L_. Compound GS-458967 was discovered by Gilead Science Inc. [43], demonstrated to be highly potent *in vitro* for block of the I_Na-L_ (Figure 4), and suppressed oxidative EAD-mediated triggered VT/VF in intact rat and rabbit hearts [13].

It is worth noticing that while oxidative stress enhances both I_Na-L_ and late I_Ca-L_, the suppression of either one, as demonstrated with roscovitine (block of the late I_Ca-L_ [39]) or with GS-458967 (block of the I_Na-L_ [40]), is sufficient to effectively suppress oxidative EAD-mediated VT/VF. This indicates possible additive or synergistic interaction between the two late inward currents in promoting the formation of EADs.

At the present, there are no clinically available Class VI gating modifier drugs as defined here, useful to the treating physician. The I_Na-L_ blocker ranolazine, approved under the trade name Ranexa^®^ for the treatment of chronic angina lacks selectivity as it also blocks the I_Kr_ and the peak I_Na_ current amplitudes, and therefore cannot be considered a member of this class. There is growing interest to develop mexiletine analogs that show high selectivity against the late but not the peak I_Na_, and initial positive experimental results are very encouraging [44].

In conclusion, the time is ripe now for the development of small molecule drugs with Class VI action (gating modifiers) to add to the traditional classes of AAD therapies. The great selectivity of Class VI drugs solely targeting arrhythmogenic currents make these gating modifiers ideal for combination therapy with traditional AADs and ICDs, and would therefore further reduce the risk of VT/VF.

It is worth noting that preliminary studies have shown that a similar dynamic scenario exists in the atria, and that block of enhanced atrial I_Na-L_ [45,46] suppresses EAD-mediated AF initiated by oxidative stress involving CaMKII activation [47,48].

Future studies should focus on discovery and development of novel and clinically safe I_Na-L_ and late I_Ca-L_ blockers, and translation of preclinical findings to clinically demonstrated benefits to patients. The goal will be to improve the currently suboptimal AAD therapy, and to provide a rational therapy against AF and VT/VF with different cardiac pathologies that are primarily due to sustained hyperactivity of CaMKII signaling. Future developments in this direction will pave the way for a move from empiricism to a more rational and targeted approach in AAD therapy.

## Figures and Tables

**Figure 1:**
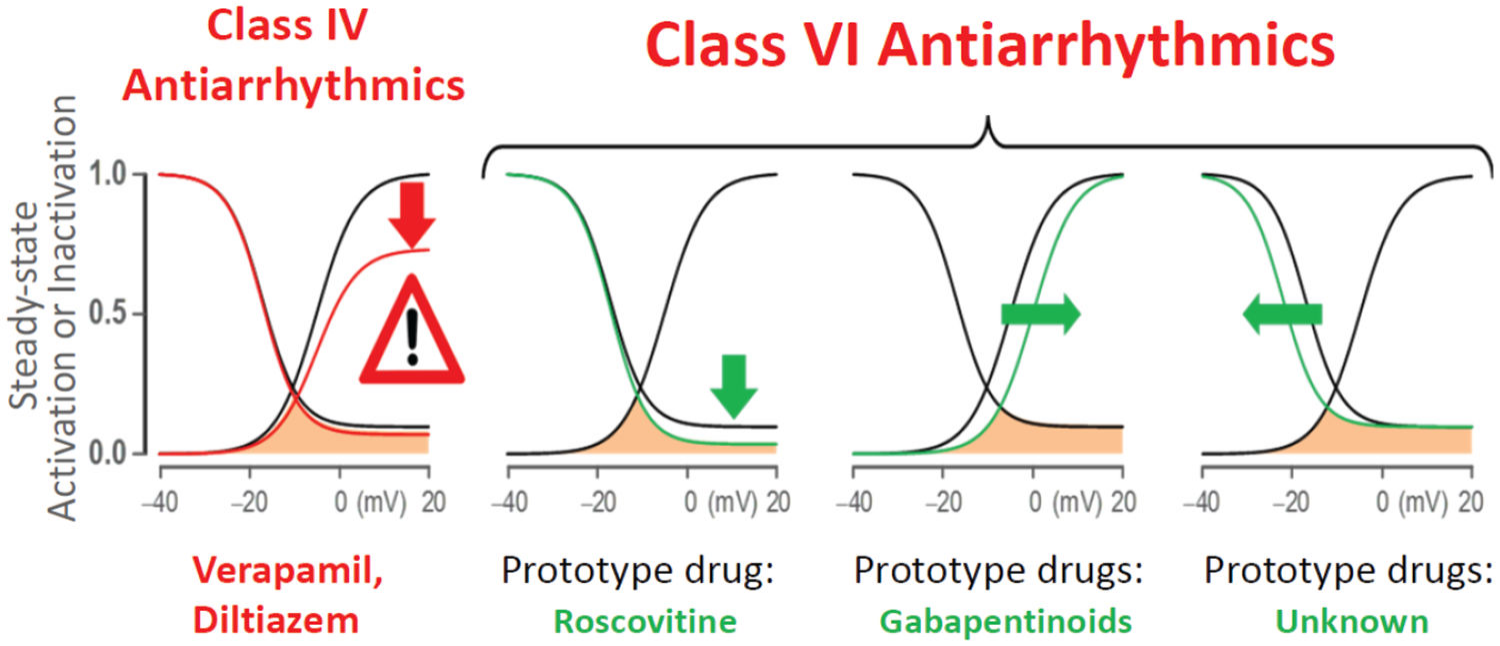
Schematic showing targets of Class VI antiarrhythmic drug action: Roscovitine, which reduces the pedestal current (downward green arrow); Gabapentinoids, which shift the steady-state activation in the depolarizing direction (rightward green arrow); potential sites of gating modifier drugs (currently no examples), which shift the steady-state inactivation curve in the hyperpolarizing direction (leftward green arrow). Similar shifts in the I_Na_ are also potential targets for AADs, however at the present no such drugs are available (see text for further discussion). Note that Class IV drugs (verapamil & diltiazem) reduce peak I_Ca-L_ (red arrow) (Figure modified from [17]).

**Figure 2:**
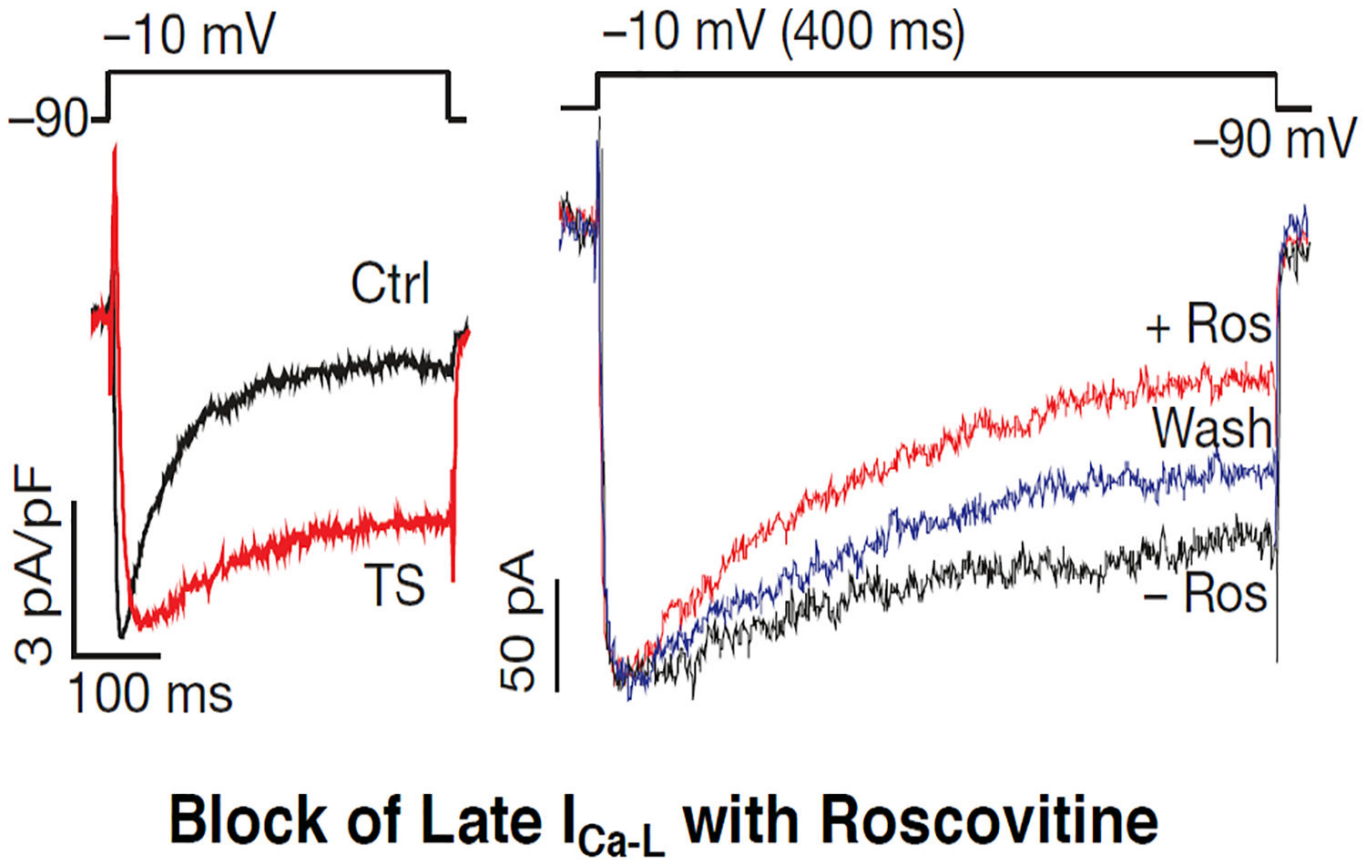
Enhanced late I_Ca-L_ in Timothy syndrome in iPSC-derived cardiomyocytes and its selective suppression by Roscovitine (Ros) without affecting peak I_Ca-L_ (modified from [29]).

**Figure 3:**
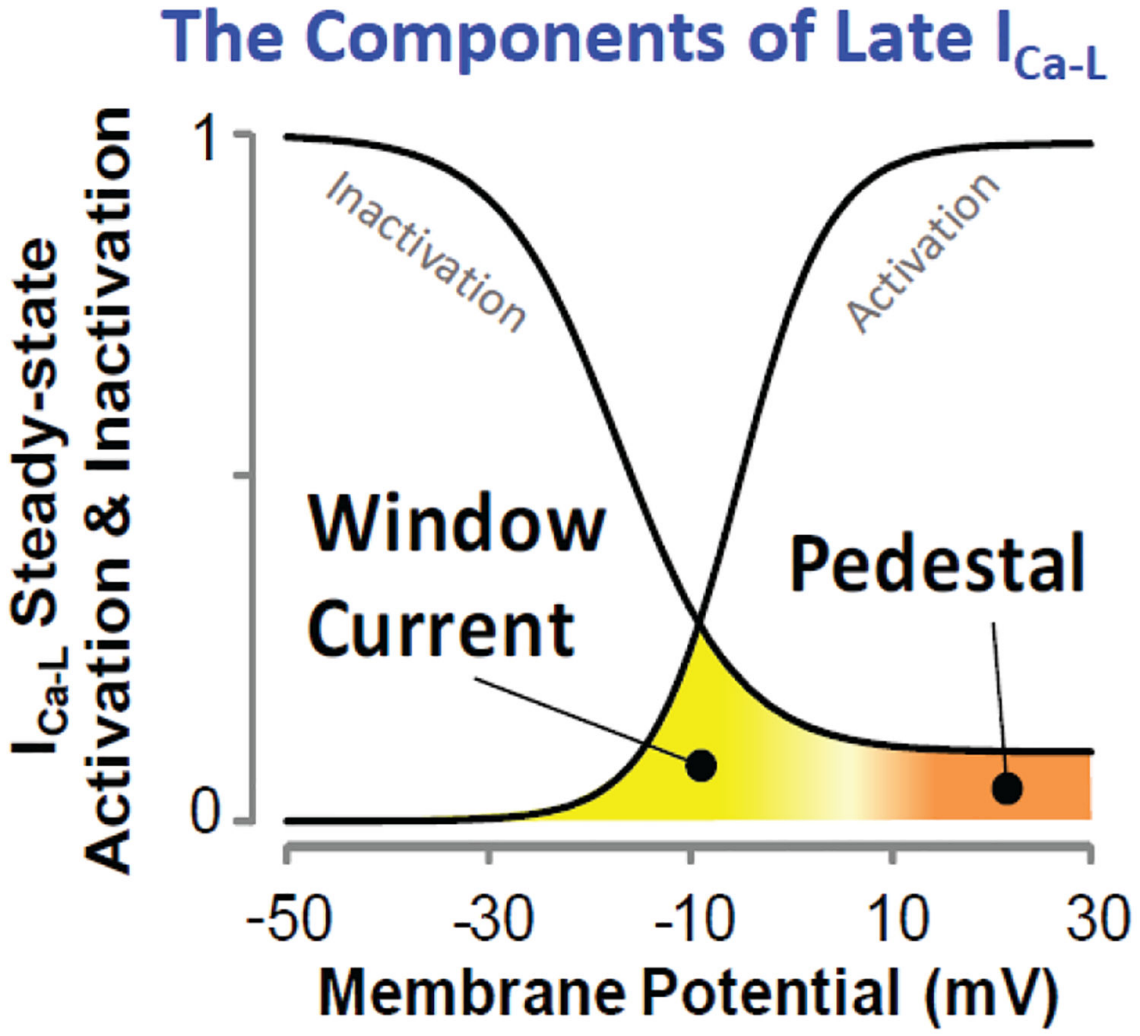
Schematic showing the components of the late I_Ca-L_, the window and pedestal current.

**Figure 4:**
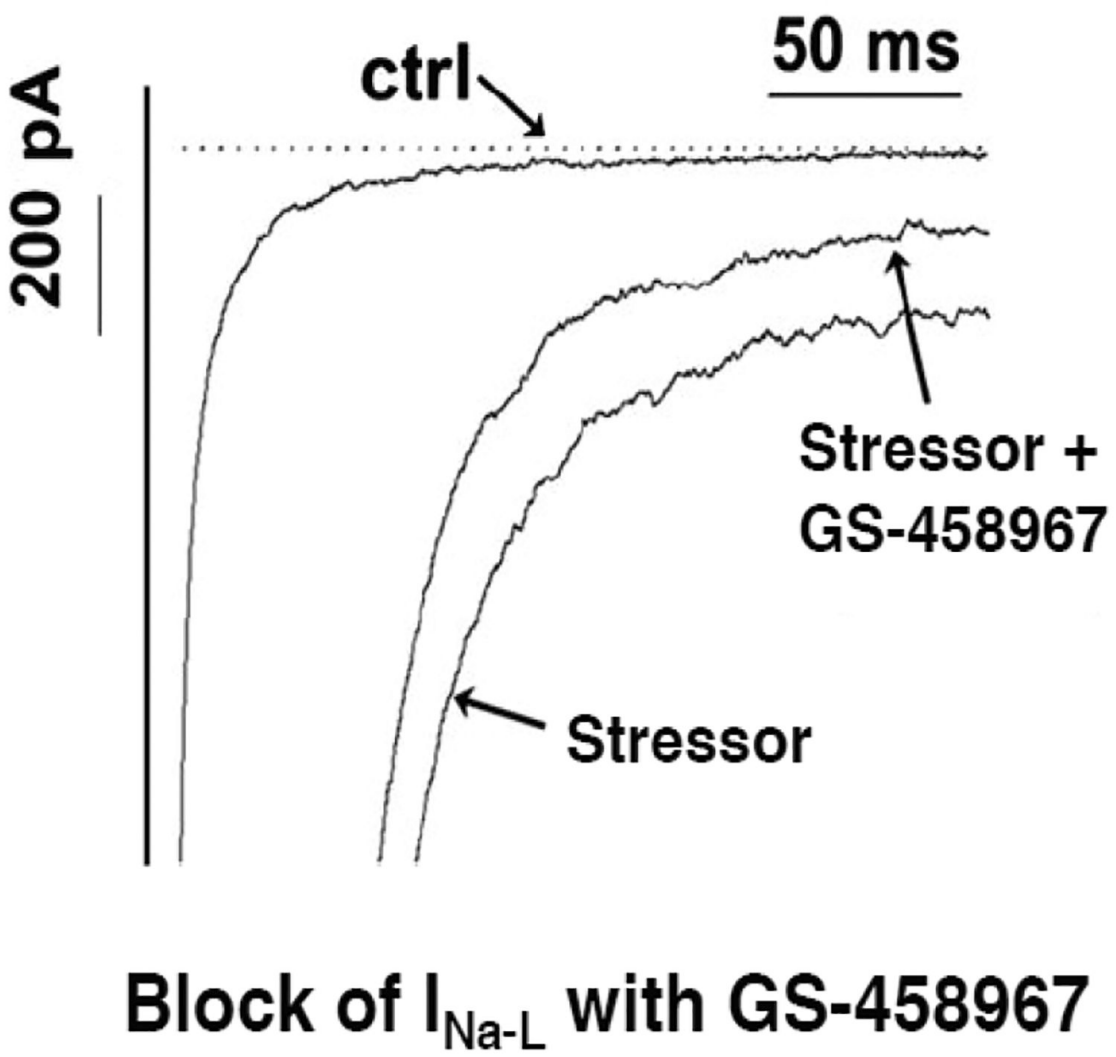
Selective suppression of the I_Na-L_ by GS-458967 in isolated cardiomyocytes (modified from [43]).
